# Synthesis and Properties of Mg-Mn-Zn Alloys for Medical Applications

**DOI:** 10.3390/ma14081855

**Published:** 2021-04-08

**Authors:** Yunpeng Hu, Delong Dong, Xiangyu Wang, Hongtang Chen, Yang Qiao

**Affiliations:** School of Mechanical Engineering, University of Jinan, Jinan 250022, China; hhyypp82516@163.com (Y.H.); 17863255295@163.com (D.D.); me_wangxy@ujn.edu.cn (X.W.); me_chenht@ujn.edu.cn (H.C.)

**Keywords:** magnesium alloys, powder metallurgy, microstructure, mechanical properties, corrosion resistance

## Abstract

The magnesium alloys Mg-0.5Mn-2Zn, Mg-1.0Mn-2Zn, and Mg-1.5Mn-2Zn (wt.%) with potential biomedical applications, synthesized by powder metallurgy, were investigated to evaluate the influence of manganese content on their microstructure, mechanical properties, and corrosion resistance. The results show that Mg-Mn-Zn alloys prepared by powder metallurgy reached the maximum compressive stress of 316 MPa and the maximum bending strength of 186 MPa, showing their good resistance to compression and bending, and meeting the mechanical properties required for the human bone plate. With an increase in manganese content, the corrosion resistance improved. In the polarization curve, the maximum positive shift of corrosion potential was 92 mV and the maximum decrease of corrosion current density was 10.2%. It was concluded that, of the alloys tested, Mg-1.0Mn-2.0Zn (wt.%) had the best overall performance, and its maximum compressive stress force and corrosion current density reached 232.42 MPa and 1.32 × 10^−5^ A·cm^−2^, respectively, being more suitable for service in human body fluids.

## 1. Introduction

In recent years, magnesium alloy as a new biodegradable biomaterial has been increasingly valued by front-line researchers in clinical applications. The greatest advantage of magnesium alloys is their excellent performance in biocompatibility and in mechanical properties. The density of magnesium and magnesium alloys is between (1.74 and 2.0 g/cm^3^) which corresponds to the density of human bones. Compared to titanium alloys and the rest of traditional medical materials, magnesium has an elastic modulus of (41–45 GPa) [1,2,3,4], which is very close to human bone, thus avoiding the adverse effects due to stress shielding. Magnesium has very good biocompatibility, belongs to the human body’s indispensable trace elements, and can achieve self-degradation in the human body, avoiding the secondary removal as a medical bone plate, as well as reducing the economic burden and physical pain of patients. However, pure magnesium has a high negative standard electrode potential (−2.37 V at 25 °C), which leads to a rapid corrosion rate of pure magnesium, especially in the human body fluid environment. At least 12 weeks are needed for the joint plate to provide mechanical support during the initial phase of the fracture to provide time for bone healing to repair the damage [5,6]. Excessive rapid degradation corrosion causes magnesium to lose its mechanical integrity before human tissue repair, while the rapid degradation of pure magnesium generates large amounts of hydrogen, leading to local pH increases in cellular tissue fluids, resulting in the possibility of cell death or tissue inflammation occurring [7]. Therefore, particularly important to prevent the rapid corrosion of magnesium by alloying it.

Powder metallurgy (PM) is a promising technique that uses the interfusion of spaced particles to prepare magnesium alloys [8]. The mechanical properties of the material it prepares can be achieved by changing the pore size and distribution within the metal alloy [9]. By controlling the size and shape of the material powder particles and by adjusting the alloy preparation conditions such as (compaction pressure, sintering time, and holding time), the pore size and distribution within the sintered alloy can be controlled [8]. Inevitably, impurities occur during the sintering process, where the melting point of the impurities may differ significantly from that of the raw material, leading to abnormal grain growth in the sintered alloy, i.e., a few areas within the slower growing fine grain matrix grow rapidly to form coarse grains, resulting in the inability to produce a neck connection between the particles, leading to excessive porosity in the sintered product, which adversely affects the ductility and compressive strength of the alloy, and reduces the overall mechanical properties of the metal alloy. Witte et al. [10] successfully prepared a composite material using AZ91D as matrix and HA (Hydroxyapatite) particles as reinforcement by powder metallurgy, and the results showed that the AZ91D/HA composite material prepared by powder metallurgy is a biomaterial that can meet the mechanical properties. Thus, considering the economic and energy consumption aspects, the powder metallurgy process is one of the most suitable techniques for preparing magnesium alloy materials [11]. Due to the good biocompatibility of zinc and manganese elements, these two elements were chosen to develop biomedical magnesium alloys in this paper. When zinc is low in the human body, almost all physiological processes are strongly disturbed. The addition of manganese leads to grain refinement of the alloy, and its most important function is that manganese removes iron and its heavy elements to improve the corrosion resistance of magnesium alloys [12]. Except for extreme occupational exposure, Mn has no toxic effects and plays a major role in the activation of several enzyme systems (hydrolases, kinases, transferases, decarboxylases, and mitochondrial respiration) [13]. Based on the above discussion, in this study, three magnesium alloys with different manganese element contents were prepared, and the microstructure, mechanical suitability, and corrosion properties of the three alloys were investigated by selecting manganese and zinc as alloying elements by powder metallurgy.

## 2. Materials and Methods

This section focuses on the material selection, preparation process, and initial microscopic morphology of the original powder, as well as the phase analysis after the preparation.

### 2.1. Materials

Table 1 shows the powder’s characteristics used for the preparation of medical Mg-Mn-Zn alloys. In order to develop medical Mg-Mn -Zn alloy with more excellent properties, spherical magnesium powder (300 mesh, purity ≥ 99.9%) was selected, in which two other different materials were added: Zn (300 mesh, purity ≥ 99.9%), Mn (300 mesh, purity ≥ 99.9%) These materials were selected from Shanghai Naio Nano Technology Co. (Shanghai, China), as shown in Table 1.

### 2.2. Preparation of Mg-Mn-Zn Alloy

Carboneras et al. [14] found that pure magnesium prepared by powder metallurgy had finer grains and outperformed the conventional casting process in terms of mechanical properties. Meanwhile, Yu et al. [15] found that the mechanical properties of 5, 10, and 15 wt.% β-Ca_3_(PO_4_)_2_/Mg-6 wt.% Zn composites prepared by powder metallurgy were consistent with the corrosion rate in human bone tissue. Therefore, the powder metallurgical process can be a potential process preparation means to manufacture biomedical materials. Thus, it is important to study whether the properties of the magnesium alloy prepared by powder metallurgy meet the human body’s needs, and the results will lay the foundation for the subsequent optimization of the preparation and processing process.

**Figure 1:**
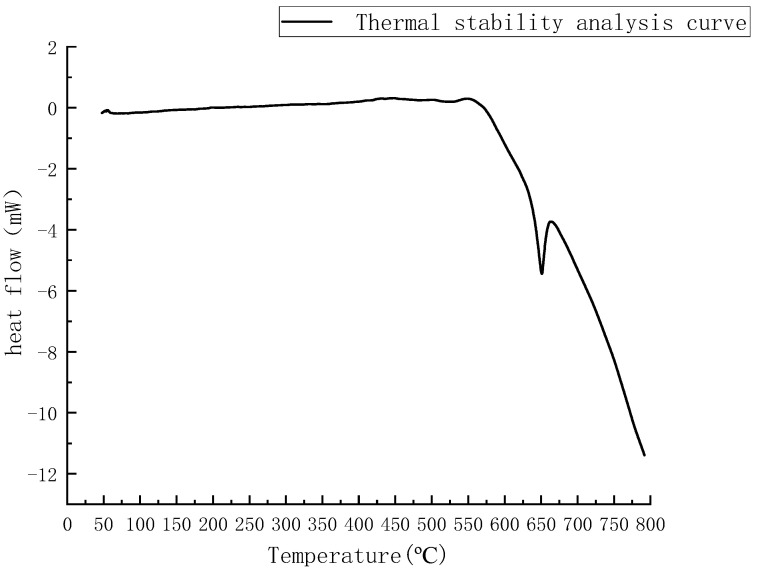
Alloy powder thermal stability analysis curve.

The target alloys sintered in this experiment were magnesium alloys with composition ratios of Mg-0.5%Mn-2%Zn, Mg-1.0%Mn-2%Zn, and Mg-1.5%Mn-2%Zn. Thermal stability analysis of the three powders was required to determine their final sintering temperatures before sintering. The powder mixture is first weighed out, using an electronic balance with a total mass of 80 g. The weighed powder is poured into the stainless steel ball-mill jar and vacuumed. Place the ball-mill jar into the planetary ball-mill machine (XQM-1-6 of Changsha Tianchuang Powder Co., Ltd., Changsha, China), with the ball-mill time set to 5 h and speed at 300 r/min. The quality ratio of ball and powders is 5:1 and intermittent ball milling is used. To prevent the steel balls inside the ball-mill jar from cold welding with the powders inside the jar due to long rotation time, the ball-mill machine stops for 10 min every 30 min of operation. Mixed powder is not a single substance, before powder metallurgy, it is necessary to obtain the temperature of the mixed powder in the solid–liquid coexistence state, and the differential scanning calorimetry is done to obtain the temperature at which the mixed powder starts to melt. The sintering temperature of the mixed powder was determined by differential scanning calorimetry (DSC) with a differential scanning calorimetry analyzer (1600HT, Mettler, Bremen, Germany) at a test temperature of 50–800 °C, using high-purity argon as the protective gas and a heating rate of 15 K/min, and the thermal stability analysis curve of the alloy powder was obtained. The maximum temperature for the sintering preparation of Mg-Mn-Zn alloy was determined to be 650 °C, as shown in Figure 1.

**Figure 2:**
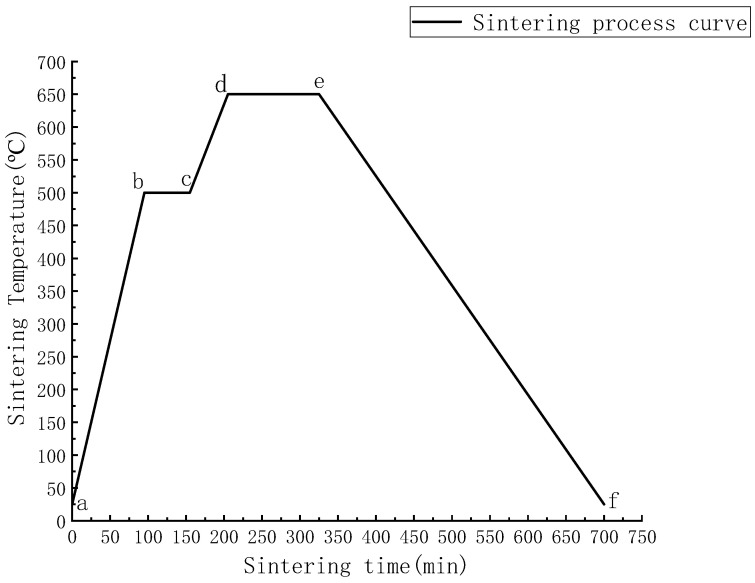
Sintering-process curve.

After determining the sintering temperature, the mixed powder was weighed out to 80 g by an electronic balance (Shanghai Sunyu Hengping Scientific Instruments Co., Ltd., Shanghai, China, FA2004) and poured into the graphite mould, which was cold-pressed at room temperature, using a universal electronic testing machine (Shenzhen New Sansi Material Testing Company CMT5305, Shenzhen, China) at a pressure of 30 kN and held under 30 kN for 5 min. The graphite mould was then placed in a vacuum sintering furnace at 5 MPa, using the two-step sintering (TSS) technique, as shown in Figure 2. In the figure, the heating rate of the ab section is 5 °C per minute, the b–c section is held at a temperature of 500 °C for 1 h, the heating rate of the cd section is 3 °C per minute, the de section is held at a temperature of 650 °C for 2 h, and the e–f section is the stage of natural cooling from 650 °C to room temperature.

### 2.3. Mechanical Performance Test

#### 2.3.1. Vickers Hardness Test

The prepared Mg-Mn-Zn alloy was processed into 10 mm × 10 mm × 10 mm specimens by wire-cutting, and the samples were decontaminated by ultrasonic cleaning, for 5 min, using an ultrasonic cleaner (Kunshan Ultrasonic Instrument KQ-100B, Suzhou, China), and polished by using 400, 800, 1000, 1200, 1500, and 2000 mesh metallographic sandpaper, step by step. The samples were polished on the grinding and polishing machine (UNIPOL-1200S of Shenyang Kejing Automation Equipment Co., Ltd., Shenyang, China). The hardness of the specimens was tested by using a digital micro hardness tester HV-1000IS (Shanghai Optical Instruments I, Shanghai, China) and a diamond Vickers indenter. The pressure applied during the test was 100 g. To ensure the accuracy of the data, each specimen was spotted 5 times on the surface, under the same conditions, and the data obtained was averaged as the hardness of the specimen.

#### 2.3.2. Compression Test

The prepared Mg-Mn-Zn alloy was made into a Ø10 mm × 18 mm compression specimen by wire-cutting. A universal electronic testing machine (Shenzhen New Sansi Material Testing Company CMT5305, Shenzhen, China) was used to carry out, at room temperature, a compression test on the metal material. In order to obtain more accurate test results, the indenter was loaded at a rate of 2 mm/min, until the test was stopped when the magnesium alloy specimen showed significant failure. Three compression tests were carried out on each compressive specimen, for accuracy, to obtain the maximum compressive strength.

#### 2.3.3. Three-Point Bending Test

A three-point bending test was carried out at room temperature, using a electronic universal testing machine (Shenzhen New Sansi Material Testing Company CMT5305, Shenzhen, China). The specimens were machined into 5 mm × 5 mm × 55 mm specimens by a wire-cutting machine.

To obtain more accurate test results, we used the bending strength equation based on the three-point bending test:

$$R=\frac{3FL}{2bh^{2}}$$

where *R* is the bending strength, *F* is the loading force, *L* is the span (40 mm), *b* is the width of the specimen (5 mm), and *h* is the height of the specimen (5 mm). A loading speed of 2 mm/min was used for the indenter, until the test was stopped when the magnesium alloy specimen failed in bending deformation. To ensure the authenticity of the three-point bending test data, all specimens were tested three times at the same indenter speed. Each specimen could only be used once.Table 2 summarizes the maximum bending strength of the specimens at different Mn contents.

### 2.4. Electrochemical Corrosion Test

Electrochemical corrosion tests were carried out, using the Shanghai C&H CHI604E electrochemical workstation (Shanghai, China). A classical three-electrode system was used for the experiments. The working electrode (Mg-Mn-Zn alloy specimen with a working area of 10 mm × 10 mm) was completely submerged—the reference electrode (saturated glycerol electrode) and the auxiliary electrode (platinum electrode) respectively. The corrosion medium was an SBF simulated body fluid to simulate the chloride concentration in the human environment. Three different ratios of Mg-Mn-Zn alloys with different Mn contents were wire-cut into 10 mm × 10 mm × 5 mm blocks for electrochemical corrosion testing. The surface to be tested was ground and polished, and the rest of the surface was covered with silica gel to ensure that the polished surface was completely submerged in the solution with a contact area of 1 cm^2^.The experimental data obtained were analyzed to obtain the best-fitting equivalent circuit model. Each test was repeated twice to increase the accuracy of the test data. Furthermore, to best simulate the environment under corrosion of human body fluids, all samples were measured at a temperature of 37 °C.

## 3. Results and Discussion

### 3.1. Original Powder Morphological Characteristics

**Figure 3:**
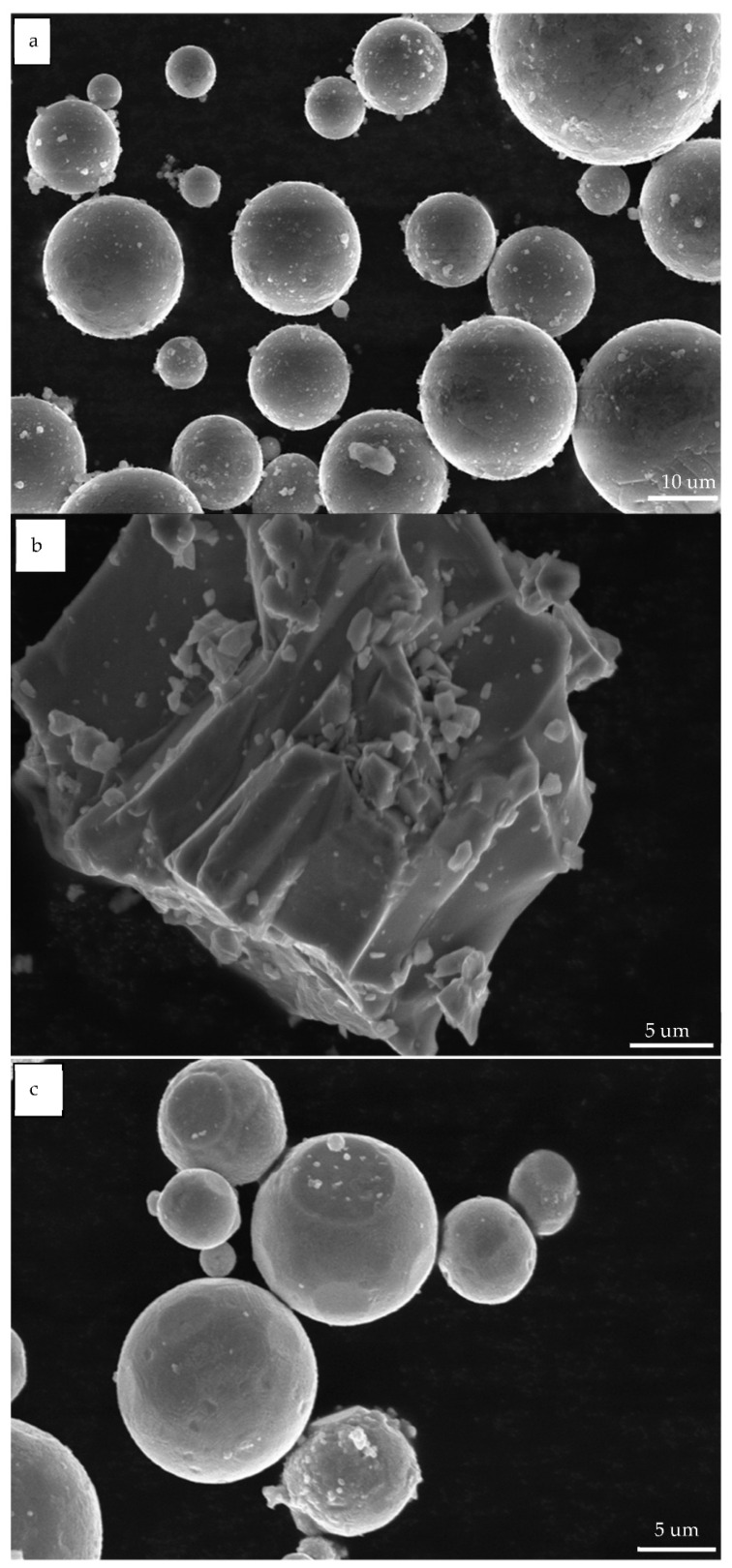
Original powder morphology of Mg-Mn-Zn material: (**a**) Mg powder, (**b**) Mn powder, and (**c**) Zn powder.

Figure 3 shows the original powder morphology of Mg-Mn-Zn material, using scanning electron microscopy (FEI USA, Inc. FEI QUANTA FEG 250, Boston, MA, USA). It can be seen that, due to the high quality required to maintain a high purity, the magnesium powder is spherical and has a spherical particle size < 40 um, with a slightly rough surface with some burrs that can be observed. Manganese powder is irregular in shape and has a size <30 um, with an angular surface profile and sharp corners. Zinc powder has a particle size of <30 um and a smoother surface than magnesium powder, characterized by a spherical shape.

**Figure 4:**
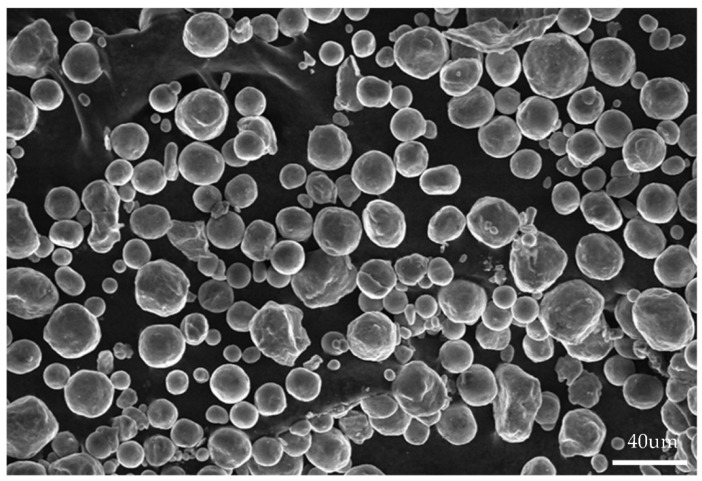
Homogeneous mixture of powders.

At the end of the ball-milling process a homogeneous mixture of powders was obtained as in Figure 4. It can be seen that, after 5 h of ball milling, the powder is well mixed, and it can be seen that the impact produces spherical magnesium-like powders of varying sizes and zinc powders, and manganese powders of varying regularity.

### 3.2. Characteristics of the Alloy Prepared by Powder Metallurgy

**Figure 5:**
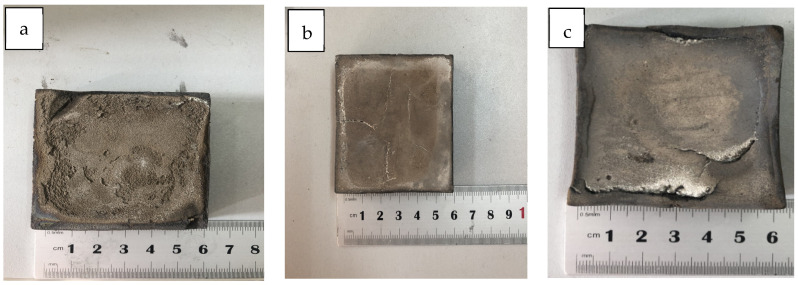
Hot-pressed magnesium alloys: (**a**) Mg-0.5%Mn-2%Zn, (**b**) Mg-1%Mn-2%Zn, and (**c**) Mg-1.5%Mn-2%Zn.

The Mg-Mn-Zn alloy material prepared by sintering is shown in Figure 5. After observation, it can be seen that the surface color of the prepared Mg-Mn-Zn alloy is basically the same and has a metallic luster, and the density is about 1.77 g/cm^3^ after measurement and calculation. In the preparation environment of hot-pressing sintering, the surface of the alloy is cracked due to the excessively high local pressure on the surface of the alloy. The reason why the prepared alloy frame is not straight is that, under natural cooling conditions, the alloy is cold when it transforms from high temperature to low temperature, which results in shrinkage.

According to reports, low-heating-rate sintering of metal specimens tends to lead to low densification of the metal, resulting in an increase in porosity of the alloy and thus a decrease in the strength of the alloy. Because the sintering temperature of many metal powder particles does not reach the melting point at low heating rate, many particles still remain dense, and the metal particles cannot generate solder joints with each other, resulting in lower density. The high heating rate can quickly reach the solder joints between the particles by rapid heating and thus promote the fusion between the metal particles, and the higher sintering temperature accelerates the diffusion of the particles and improves the densification of the metal alloy [16,17].

### 3.3. Mechanical Property Analysis

#### 3.3.1. Microhardness and Bending Force

**Figure 6:**
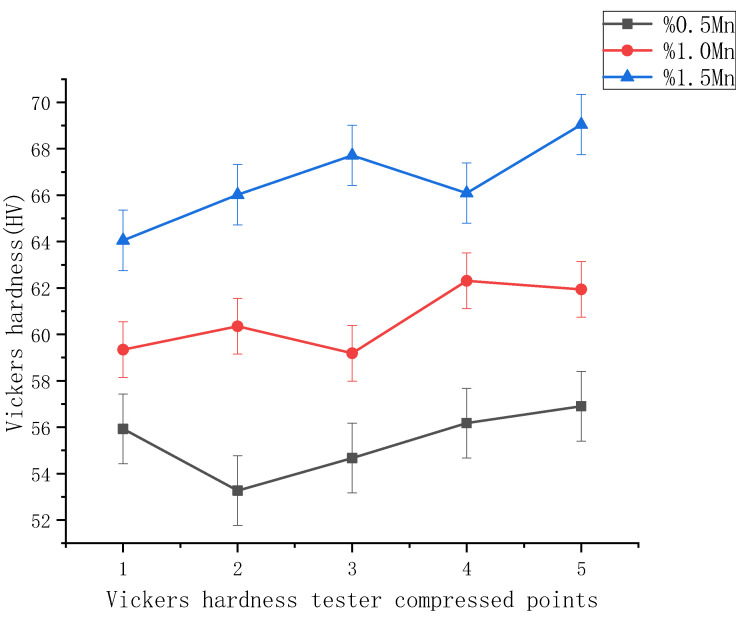
Hardness curves of the three alloys.

The hardness curves of the three alloys (Figure 6) show that the hardness of these three alloys increases as the manganese content increases. The hardness of the Mg-0.5%Mn-2%Zn alloy reached 56 HV, the Mg-1.0%Mn-2%Zn alloy reached 62 HV, and the Mg-1.5%Mn-2%Zn alloy reached 69 HV. The possible reason for this is that, by adding manganese, it serves to refine the grain of the alloy, which in turn improves the surface integrity of the whole alloy. Therefore, alloying magnesium with manganese can effectively increase the hardness of magnesium alloys.

**Figure 7:**
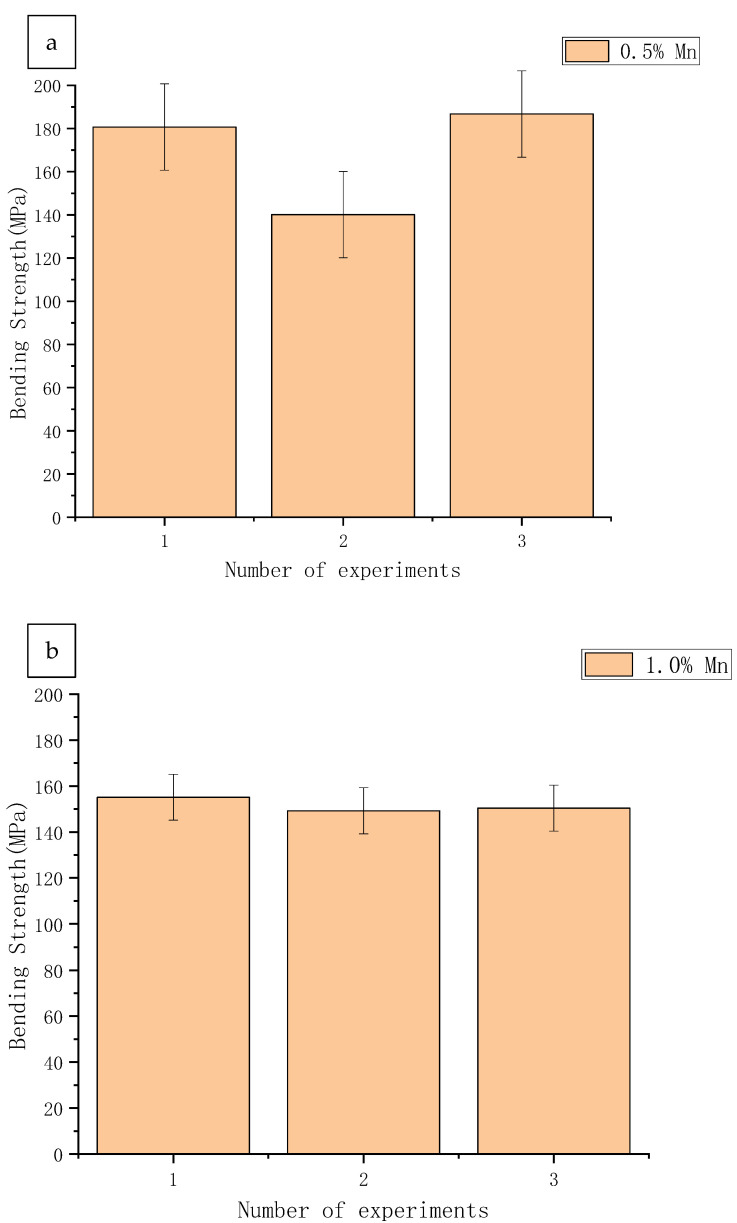

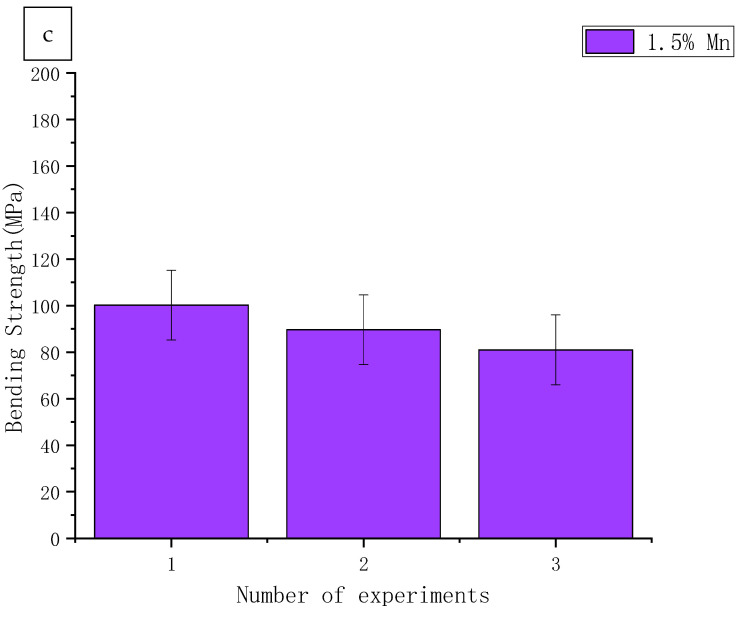
Maximum bending strength of the specimen. (**a**) 0.5% Mn; (**b**) 1.0% Mn; (**c**) 1.5% Mn.

Figure 7 plots the maximum bending strength of the sample alloy in the three-point bending test under the same movement rate displacement of the indenter, in which the maximum bending strength of the three alloys are marked. With the change of displacement, the bending force keeps increasing and the bending strength also increases gradually, and it can be seen that the maximum bending strength also changes according to the increase of manganese content, that is, the maximum bending force decreases with the increase of manganese content. The reason may be that, with the increase of manganese content, the plasticity of the alloy starts to decay and, thus, the brittleness increases, which leads to the decrease of the bending strength of the material. This is due to the fact that the increase in manganese content causes dynamic recrystallization during the preparation process, which leads to a decrease in the flexural and compressive strength of the alloy [18].

#### 3.3.2. Compression Force

**Figure 8:**
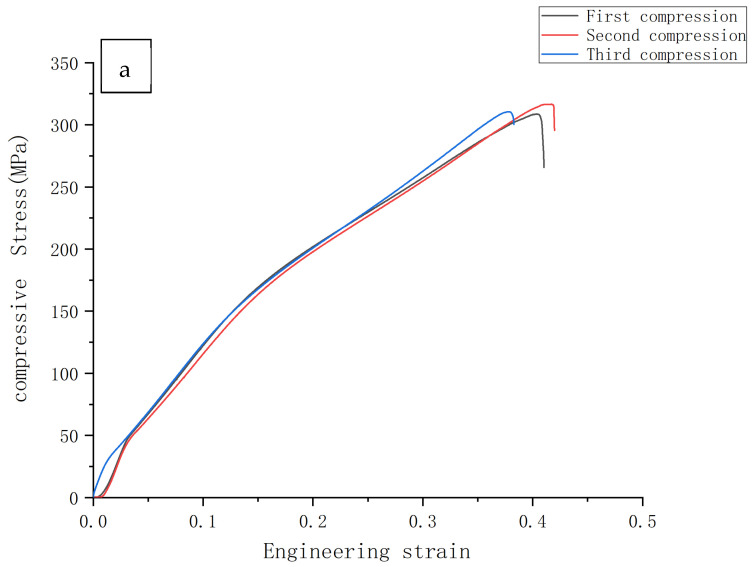

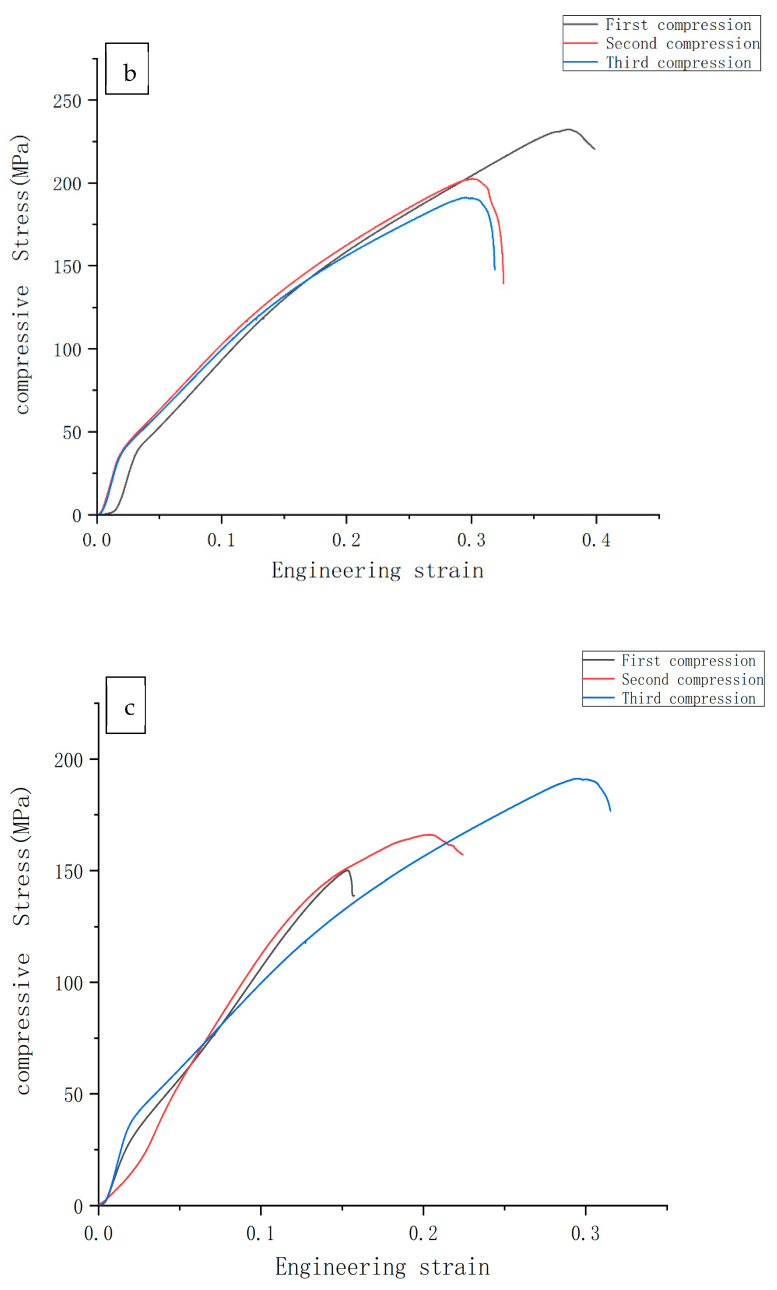
Maximum compressive stress: (**a**) Mg-0.5%Mn-2%Zn, (**b**) Mg-1%Mn-2%Zn, and (**c**) Mg-1.5%Mn-2%Zn.

Table 2 and Table 3 show the mechanical properties of the specimens under three-point bending test and compression test, respectively. The compression test also further verifies that the maximum compressive stress changes with the increase of manganese content. The phenomenon is that the maximum compressive stress of the alloy becomes smaller as the content of manganese element increases. At the same time, it can be inferred that, when the content of manganese element increases, the plasticity of the alloy continues to decrease and the brittleness continues to increase. In order to show more visually the maximum compressive stress of the alloy at different Mn contents, the maximum compressive stress of the specimen is shown in Figure 8.

Table 3 shows that the maximum compressive stress of the three magnesium alloys with different manganese content can reach up to 316.61 MPa at 0.5% of manganese element and only 166.03 Mpa at 1.5%.

### 3.4. Microstructure Characterization after Powder Metallurgy Preparation

The sintered Mg-Mn-Zn alloy was cut into 10 mm × 10 mm × 10 mm pieces, using a wire cutter prior to microscopic observation of the specimens. The specimens were grinded with 400–2000 grit sandpaper and then polished with diamond paste until a metallic-mirror finish was achieved, and the oxidation layer was removed by using a nitric acid solution (2%).

**Figure 9:**
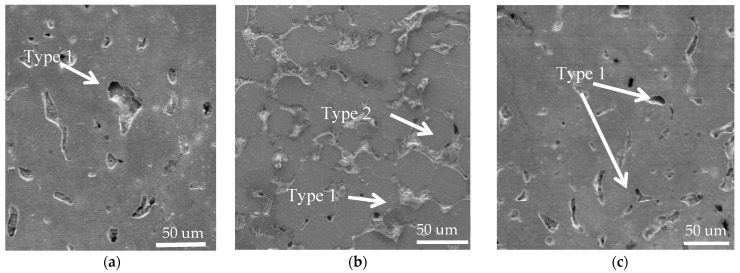
Surface morphology of the alloy (**a**) Mg-0.5%Mn-2%Zn, (**b**) Mg-1%Mn-2%Zn, and (**c**) Mg-1.5%Mn-2%Zn.

The cross-section of the Mg-Mn-Zn alloy specimen was observed by using a scanning electron microscope (FEI USA, Inc., FEI QUANTA FEG 250, Boston, MA, USA), as shown in Figure 9. It can be seen that the surface morphology of the alloy has a uniform texture and no elemental segregation. With the increase of manganese element content, it can be seen that the pores on the surface of the alloy are reduced and the pore diameter is decreased. It shows that manganese can play the role of grain refinement and can reduce the generation of pores. Figure 9 shows that the metal alloys prepared by powder metallurgy have more pores and porosity on the cross-sectional surface, and such pores and porosity can be basically grouped into two main categories. Type 1 holes are 20–30 um in diameter and have a narrow, irregular shape. The main reason for the formation of such holes is that, during the sintering process, part of the surface of magnesium particles is still covered by a thin layer of oxide. Because oxygen has a close affinity with magnesium, the magnesium reacts with the gas in the pores during the sintering process, and local combustion occurs on the surface of the magnesium particles. In the above process, because of the uneven transfer of pressure, the phenomenon of incomplete compaction exists. The local combustion produced gas expansion, so it led to the creation of holes. The Type 2 pores are generated because the magnesium powder purchased for this hot-pressing sintering is spherical magnesium powder. In the microscopic state, there are voids when the spherical magnesium powder is stacked on each other. The sintering temperature may be slightly lower or the pressure may be less, resulting in such naturally occurring voids that are not eliminated by the sintering preparation.

#### Fracture Morphology Analysis

**Figure 10:**
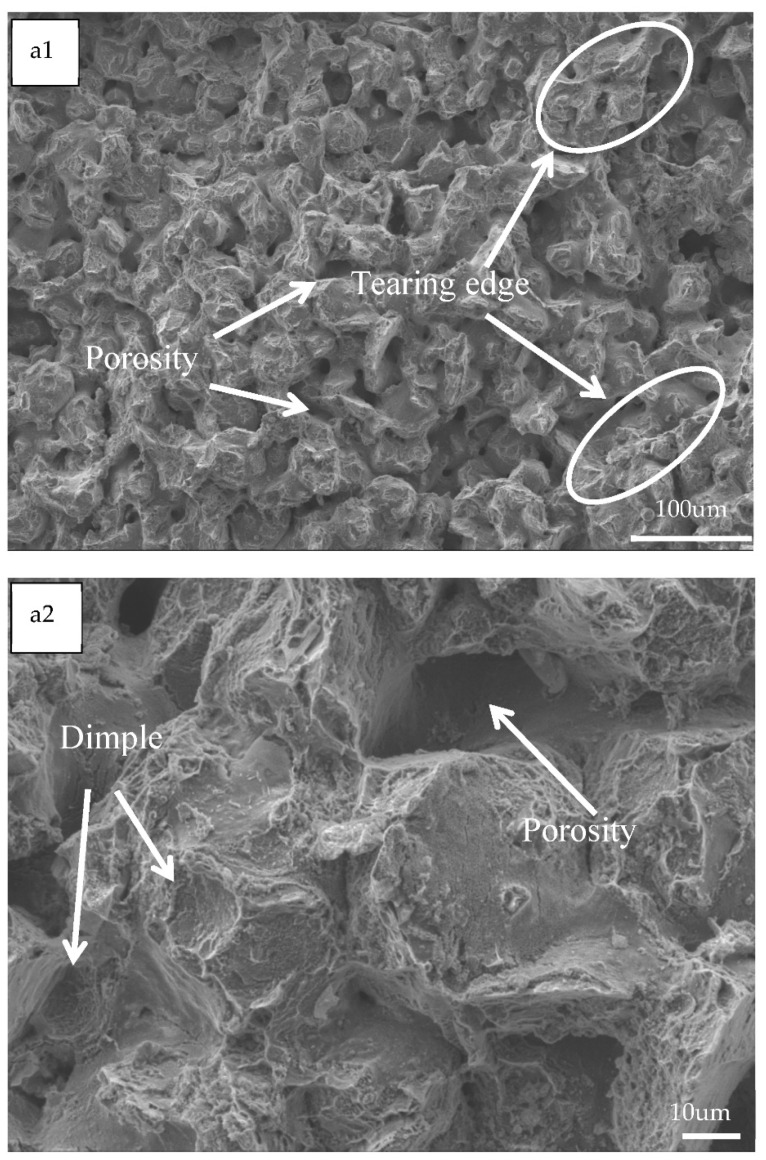

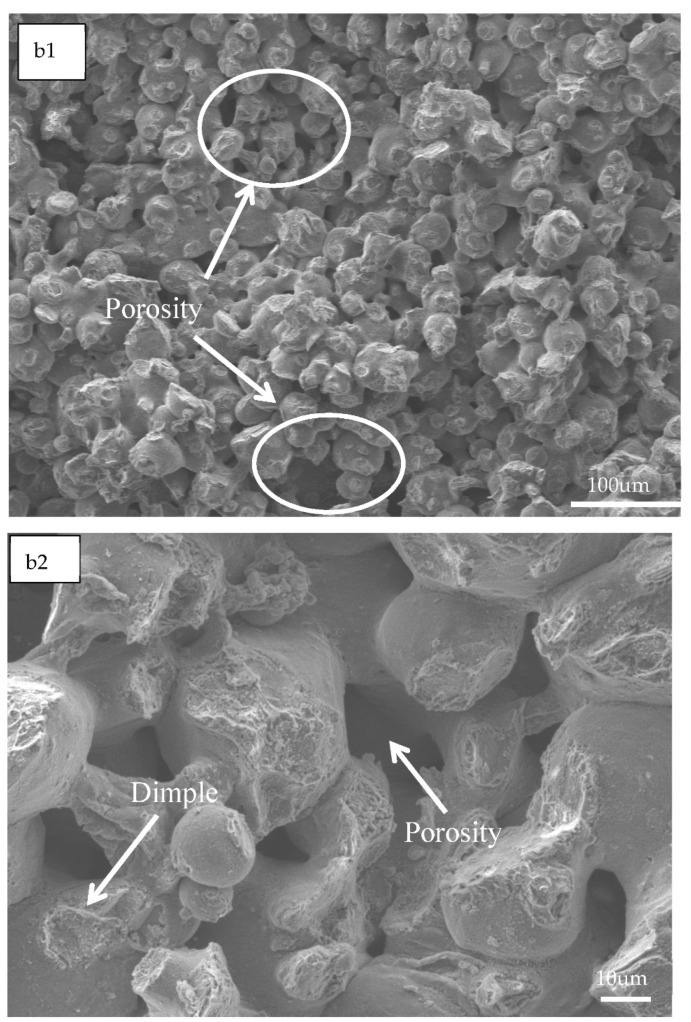

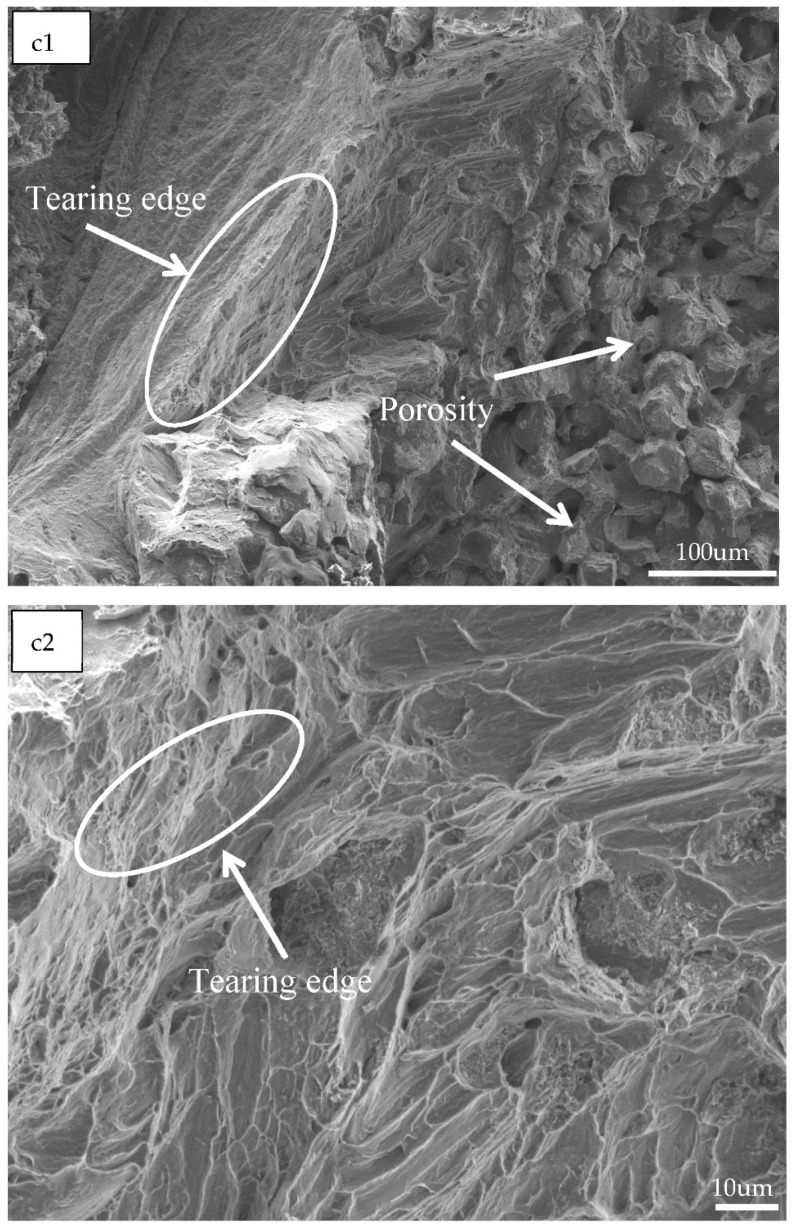
Fracture morphology of alloy (**a1**,**a2**) Mg-0.5%Mn-2%Zn, (**b1**,**b2**) Mg-1.0%Mn-2%Zn, and (**c1**,**c2**), Mg-1.5%Mn-2%Zn.

The fracture surfaces of the specimens at the end of the bending test are shown in Figure 10. The fracture surface of the alloy can be seen in the electron microscope images at 200× and 1000× magnification, respectively, which show a large number of brittle fracture traces, as can be seen in Figure 10a1,a2,b1,b2,c1,c2. The fracture shape starts to show a radial tearing shape due to the extremely rapid expansion of the crack, which has the characteristics of a brittle fracture [15]. However, the plastic deformation is still visible after magnification, as the specimen is subjected to a large amount of force within the alloy in the early stages of bending deformation, resulting in a large amount of plastic deformation between the internal metal particles, such as the presence of a large number of tough nests on the fracture surface that are more pronounced with plastic deformation at 0.5% manganese addition. With increasing manganese, the microscopic morphology of the bending fracture shows that the alloy generally exhibits localized tough nests with bending tearing ribs and a few areas of fracture along the grain. The brittle fracture of the alloy is more pronounced at high levels of manganese.

### 3.5. Physical Phase Analysis

**Figure 11:**
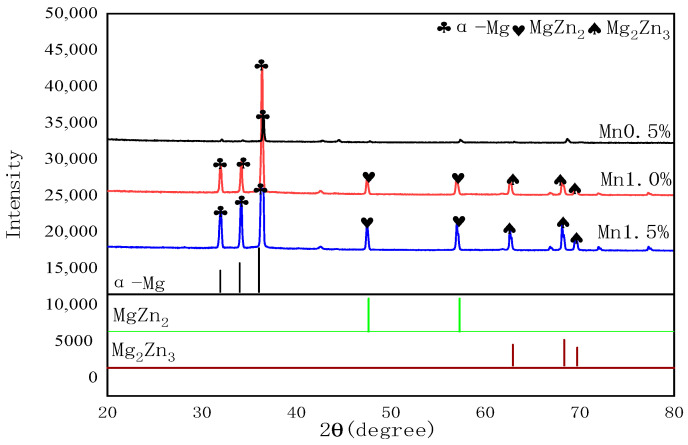
XRD phase composition analysis of Mg-Mn-Zn alloy.

The XRD pattern of Mg-Mn-Zn prepared after hot-press sintering was obtained by using CuKα source X-ray diffraction (XRD, JDX-8030, Jeol, Osaka, Japan). The XRD device was operated under a CuKα (λ = 0.1541 nm) radiation, the 2θ range of 20°−80° with a step size of 0.02°, a measurement time per step of 0.2 s, and an Ni filter. As shown in Figure 11, the identification of all reflections was done using jade software.

The intermetallic phases such as Mg_2_Zn_3_, and MgZn_2_ appear as peaks in the diffractograms, resulting in eutectic transformation during the sintering process, and these intermetallic phases can improve the corrosion resistance of Mg [19,20], and the highest peak in the α-Mg matrix is the main phase produced from the elemental phase composition prepared. Since the solid solution of manganese in the magnesium matrix is limited, no intermetallic compound is formed between magnesium and manganese, and it is also possible that the manganese content added to this Mg-Zn alloy is so small that it does not produce a great change in the phase organization, resulting in no independent phase generation of manganese; thus, no α-Mn phase peak is observed in the XRD analysis. The different color lines can distinctly represent the different component content and the different phase

### 3.6. Electrochemical Analysis

**Figure 12:**
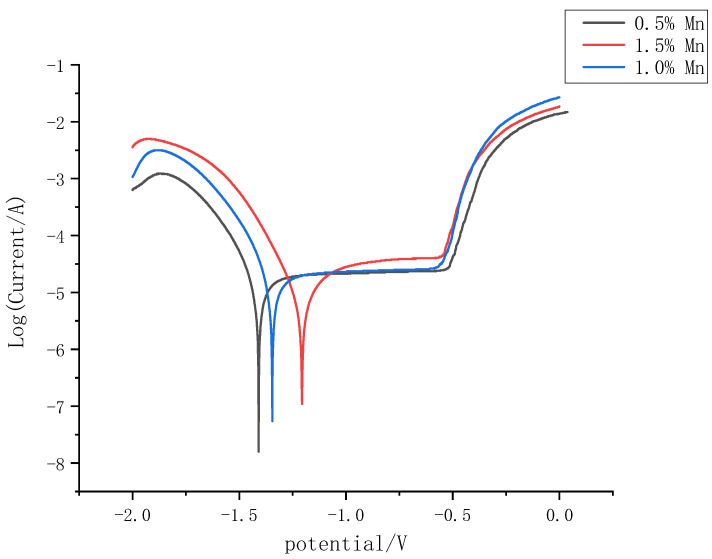
Potentiodynamic polarization curve of Mg-Mn-Zn alloy.

The electrochemical SBF of the three Mg-Mn-Zn alloys prepared after powder metallurgy simulates the Tafel polarization curves in human body fluids, as shown in Figure 12. The corrosion parameters (corrosion potential, φ_corr_, and corrosion current density, J_corr_) for the three Mg-Mn-Zn alloys obtained from the Tafel polarization curves are shown in Table 4. The results show that the alloys with high Mn content have superior corrosion resistance compared to the alloys with less Mn content added. The reason for this is that the addition of Mn reduces the corrosion rate of the Mg-Zn alloy. Mn forms similar oxides such as MnO_2_ and MnO by reacting with the phosphate in the SBF solution, and such oxides are a protective film similar to the type of conversion film [21], as indicated by the polarization curves; with increasing Mn content, the corrosion potential moves to a higher potential. Such protective films cover the grain-boundary surface, reducing the corrosion rate, delaying the breakdown and pitting of the protective film, and improving the stability of the surface corrosion product film [22], which significantly inhibits the corrosion of magnesium alloys; thus, the addition of Mn reduces the corrosion rate of the alloy.

## 4. Conclusions

In this study, the Mg-Mn-Zn alloy was successfully prepared by powder metallurgy, and in this paper, its microstructure, mechanical properties, and corrosion resistance were analyzed and following conclusions were drawn:

1. From the results, in the microstructure, as the content of manganese increases, it can be seen that the porosity of the alloy cross-section decreases and the diameter of the pores decreases. This indicates that the manganese element can have a grain-refining effect and reduce the generation of pores. In the fracture morphology, it can be seen that, as the manganese content increases, tearing edge representatives of brittle fracture are observed.
2. The results show that the mechanical properties of the Mg-Mn-Zn alloy prepared by powder metallurgy have a maximum compressive stress of 316 MPa and a maximum bending strength of 186 MPa. The microhardness of the Mg-Mn-Zn alloy prepared after powder metallurgy increased with increasing manganese content, and the hardness values of Mg-1.5Mn-2Zn (wt.%) were effectively increased by 20% compared to those of Mg-0.5Mn-2Zn (wt.%).
3. From the electrochemical corrosion experiments, with the increase of manganese content in the Mg-Mn-Zn alloy, the corrosion resistance of the alloy was improved, and in the polarization curve, the maximum positive shift of the corrosion potential of the specimens was 92 mv, and the maximum decrease of the corrosion current density was 10.2%. By comparing the above properties, it was concluded that Mg-1.0Mn-2.0Zn (wt.%) had the best overall performance among the three alloys prepared, and its maximum compressive stress and corrosion current density reached 232.42 MPa and 1.32 × 10^−5^ A·cm^−2^, respectively. Therefore, it is more suitable for service in the human body fluid environment.

## Figures and Tables

**Table 1:** Powder material parameters for the preparation of medical Mg-Mn-Zn alloy.

Powder	Powder Shape	Particle Size/μm	Purity/wt.%	Melting Point/°C
Mg	Spherical	<40	99.9	648.9
Mn	Irregular shape	<30	99.9	1244
Zn	Spherical	<30	99.9	419.5

**Table 2:** Bending strength of Mg-Mn-Zn alloy.

Magnesium Alloy	Maximum Bending Force (kN)	Maximum Bending Strength (MPa)
Mg-0.5%Mn-2%Zn	389.05 ± 12	186.74 ± 20
Mg-1.0%Mn-2%Zn	256.49 ± 8.5	155.11 ± 10
Mg-1.5%Mn-2%Zn	175.08 ± 10.5	101.06 ± 15

**Table 3:** Compression test of Mg-Mn-Zn alloy.

Magnesium Alloy	Maximum Compression Force (kN)	Compressive Strength (Mpa)	Compression Ratio to Specimen (%)
Mg-0.5%Mn-2%Zn	24.866 ± 0.645	316.61 ± 3.951	41.98
Mg-1.0%Mn-2%Zn	18.253 ± 1.618	232.42 ± 20.60	32.57
Mg-1.5%Mn-2%Zn	14.570 ± 1.437	185.611 ± 18.30	24.50

**Table 4:** Parameters of the Tafel polarization curve of Mg-Mn-Zn alloy.

Magnesium Alloy	φ_corr_/V	J_corr_/(A·cm^−2^)
Mg-0.5%Mn-2%Zn	−1.409	1.46 × 10^−5^
Mg-1.0%Mn-2%Zn	−1.345	1.32 × 10^−5^
Mg-1.5%Mn-2%Zn	−1.187	1.31 × 10^−5^

## Data Availability

The data presented in this study are available on request from the corresponding author.

## References

[1] Raftopoulos D., Katsamanis E., Saul F., Liu W., Saddemi S. (1993). An intermediate loading rate technique for the determination of mechanical properties of human femoral cortical bone. J. Biomed. Eng..

[2] Mert F. (2017). Wear behaviour of hot rolled AZ31B magnesium alloy as candidate for biodegradable implant material. Trans. Nonferr. Met. Soc. China.

[3] Lukyanova E., Anisimova N., Martynenko N., Kiselevsky M., Dobatkin S., Estrin Y. (2018). Features of in vitro and in vivo behaviour of magnesium alloy WE43. Mater. Lett..

[4] Cao F., Song G.-L., Atrens A. (2016). Corrosion and passivation of magnesium alloys. Corros. Sci..

[5] Du H., Wei Z., Liu X., Zhang E. (2011). Effects of Zn on the microstructure, mechanical property and bio-corrosion property of Mg–3Ca alloys for biomedical application. Mater. Chem. Phys..

[6] Gu X.-N., Zheng Y.-F. (2010). A review on magnesium alloys as biodegradable materials. Front. Mater. Sci. China.

[7] Witte F., Hort N., Vogt C., Cohen S., Kainer K.U., Willumeit R., Feyerabend F. (2008). Degradable biomaterials based on magnesium corrosion. Curr. Opin. Solid State Mater. Sci..

[8] Hao G.L., Han F.S., Li W.D. (2008). Processing and mechanical properties of magnesium foams. J. Porous Mater..

[9] Wang H., Jiang Q., Wang Y., Ma B., Zhao F. (2004). Fabrication of TiB2 particulate reinforced magnesium matrix composites by powder metallurgy. Mater. Lett..

[10] Witte F., Feyerabend F., Maier P., Fischer J., Störmer M., Blawert C., Dietzel W., Hort N. (2007). Biodegradable magnesium–hydroxyapatite metal matrix composites. Biomaterial.

[11] Kang S., Ebrary I. (2014). Sintering: Densification, grain growth, and microsturcture. J. Phys. Colloq..

[12] Zander D., Zumdick N.A. (2015). Influence of Ca and Zn on the microstructure and corrosion of biodegradable Mg−Ca−Zn alloys. Corros. Sci..

[13] Walton T. (1998). Biomaterials metals and alloys web alert. Curr. Opin. Solid State Mater. Sci..

[14] Carboneras M., Hernández L., Del Valle J., Garcia-Alonso M.C., Escudero M.L. (2010). Corrosion protection of different environmentally friendly coatings on powder metallurgy magnesium. J. Alloy. Compd..

[15] Yu K., Chen L., Zhao J., Li S., Dai Y., Huang Q., Yu Z. (2012). In vitro corrosion behavior and in vivo biodegradation of biomedical β-Ca3(PO4)2/Mg–Zn composites. Acta Biomater..

[16] Padmavathi C., Upadhyaya A., Agrawal D. (2011). Effect of microwave and conventional heating on sintering behavior and properties of Al–Mg–Si–Cu alloy. Mater. Chem. Phys..

[17] Lóh N., Simão L., Faller C., De Noni A., Montedo O. (2016). A review of two-step sintering for ceramics. Ceram. Int..

[18] Liu C., Liu Z. (2006). Research progress on dynamic recrystallization of magnesium and magnesium alloys. Chin. J. Nonferrous Met..

[19] Zhang W., Fan J., Zhang H. Grain Refinement and Mechanical Properties of High Pressure Low Temperature Sintered AZ91 Magnesium Alloy. Proceedings of the 11th National Conference of Heat Treatment.

[20] Yin P., Li N.F., Lei T., Liu L., Ouyang C. (2013). Effects of Ca on microstructure, mechanical and corrosion properties and biocompatibility of Mg–Zn–Ca alloys. J. Mater. Sci. Mater. Med..

[21] Jiang M., Xu C., Nakata T., Yan H., Chen R., Kamado S. (2016). High-speed extrusion of dilute Mg-Zn-Ca-Mn alloys and its effect on microstructure, texture and mechanical properties. Mater. Sci. Eng. A.

[22] Cho D.H., Lee B.W., Park J.Y., Cho K.M., Park I.M. (2017). Effect of Mn addition on corrosion properties of biodegradable Mg-4Zn-0.5Ca-xMn alloys. J. Alloy. Compd..

